# Concurrent detection of autolysosome formation and lysosomal degradation by flow cytometry in a high-content screen for inducers of autophagy

**DOI:** 10.1186/1741-7007-9-38

**Published:** 2011-06-02

**Authors:** Phillip Hundeshagen, Anne Hamacher-Brady, Roland Eils, Nathan R Brady

**Affiliations:** 1 Division of Theoretical Bioinformatics, German Cancer Research Center and Institute of Pharmacy and Molecular Biotechnology, Bioquant, University of Heidelberg, Heidelberg, Germany; 2 Systems Biology of Cell Death Mechanisms, German Cancer Research Center, Bioquant, Heidelberg, Germany; 3 Department of Surgery, Medical Faculty, University of Heidelberg, Heidelberg, Germany

## Abstract

**Background:**

Autophagy mediates lysosomal degradation of cytosolic components. Recent work has associated autophagic dysfunction with pathologies, including cancer and cardiovascular disease. To date, the identification of clinically-applicable drugs that modulate autophagy has been hampered by the lack of standardized assays capable of precisely reporting autophagic activity.

**Results:**

We developed and implemented a high-content, flow-cytometry-based screening approach for rapid, precise, and quantitative measurements of pharmaceutical control over autophagy. Our assay allowed for time-resolved individual measurements of autolysosome formation and degradation, and endolysosomal activities under both basal and activated autophagy conditions. As proof of concept, we analyzed conventional autophagy regulators, including cardioprotective compounds aminoimidazole carboxamide ribonucleotide (AICAR), rapamycin, and resveratrol, and revealed striking conditional dependencies of rapamycin and autophagy inhibitor 3-methyladenine (3-MA). To identify novel autophagy modulators with translational potential, we screened the Prestwick Chemical Library of 1,120 US Food and Drug Administration (FDA)-approved compounds for impact on autolysosome formation. In all, 38 compounds were identified as potential activators, and 36 as potential inhibitors of autophagy. Notably, amongst the autophagy enhancers were cardiac glycosides, from which we selected digoxin, strophanthidin, and digoxigenin for validation by standard biochemical and imaging techniques. We report the induction of autophagic flux by these cardiac glycosides, and the concentrations allowing for specific enhancement of autophagic activities without impact on endolysosomal activities.

**Conclusions:**

Our systematic analysis of autophagic and endolysosomal activities outperformed conventional autophagy assays and highlights the complexity of drug influence on autophagy. We demonstrate conditional dependencies of established regulators. Moreover, we identified new autophagy regulators and characterized cardiac glycosides as novel potent inducers of autophagic flux.

## Background

Macroautophagy (hereafter referred to as autophagy), the process of cytoplasmic component degradation via lysosomes, has a multifaceted involvement in human disease, including neurodegeneration, viral and bacterial infections, heart disease, and cancer [1,2]. Positive and negative control of autophagic activity is distributed among signaling pathways involved in a wide range of stress and survival responses [3-5]. Intriguingly, compounds activating autophagy via the AMP-activated protein kinase (AMPK)/mammalian target of rapamycin (mTOR) pathway, including resveratrol and rapamycin, exert protective effects in models of cardiovascular disease, but cytotoxic or cytostatic effects in cancer models [6-8]. Given its high degree of integration into major cell signaling pathways, autophagy represents an attractive target for pharmaceutical manipulation.

Autophagy is a dynamic process which can be classified into three discrete stages: (1) sequestration of cytosolic components by the autophagosome, (2) fusion of the autophagosome with the lysosome to form the autolysosome, and (3) degradation of autophagosomal contents by proteases within the lysosome. Moreover, the endosomal pathway is highly integrated into the autophagosomal and lysosomal pathways. Late endosomes undergo fusion with lysosomes and autophagosomes [9], and the endosomal sorting complexes mediate autolysosome formation [10-12].

High-content screening for the identification of small compounds to regulate autophagy is limited by the lack of methods to specifically quantify each step of the autophagy process. This, however, is the prerequisite for the robust interpretation of autophagic activity. Recent studies utilized fluorescence detection of green fluorescent protein-microtubule-associated protein 1 light chain 3 B (GFP-LC3) vesicles [13,14], specific autophagy substrates [15], or luciferase-based assays [16] for inferring activities. However, these assays are restricted to individual steps of the autophagic pathway and do not allow for concurrent monitoring of multiple steps within the autophagic/endolysosomal process.

A robust screen must identify compounds that specifically target the events within the autophagic or endolysosomal pathway, as they share many common cellular regulatory mechanisms [12]. In addition, it is of interest to compare relative drug effects obtained under different settings, including conditions, time points, and concentrations. Here, we sought to identify the impact of compounds on autophagic activity using fluorescent protein-based sensors for autophagic and endolysosomal activities. We used (i) GFP-LC3 [17] to quantify autolysosome formation, (ii) mCherry-GFP-LC3 [18] (tandem-LC3) to simultaneously monitor autolysosome formation and degradation events, and (iii) GFP-Rab7 [19,20] as a marker of general changes in endolysosomal activities. As a screening platform we utilized flow cytometry, which allows for multiparametric and quantitative detection with high sampling rates, and the generation of results amenable to statistical analysis. Importantly, the integration of multiple pathway sensors by flow cytometry allowed for the precise quantification of autophagic flux without the need for lysosomal inhibitors. Automated sampling in 96-well plates was used for measuring time-dependent changes in autophagic and endolysosomal activities. Following pipeline validation, using commonly used drug modulators of autophagy, we screened the Prestwick Chemical Library (http://www.prestwickchemical.com), consisting of 1,120 US Food and Drug Administration (FDA)-approved compounds, for modulators of autophagy. We demonstrate that lysosomal-inhibitor independent, multiparametric screening outperforms conventional autophagy assays, and we identified and validated cardiac glycosides as novel potent and specific enhancers of autophagic flux.

## Results

### Flow cytometry detection of autolysosome formation and degradation using tandem-LC3 overcomes requirement for lysosomal inhibitors to infer autophagic flux

**Figure 1:**
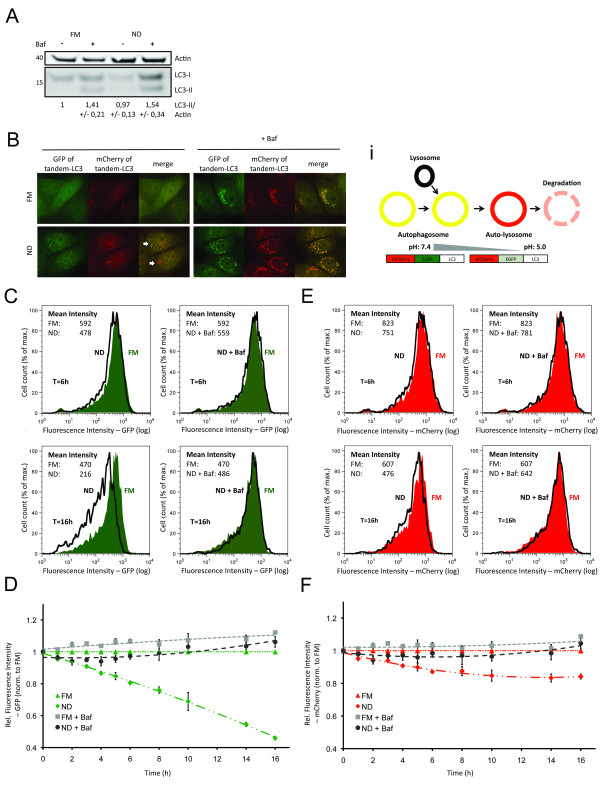
**Quantitative detection of autophagosomal formation and degradation using flow cytometry**. **(a) **Western blot analysis of wild-type cells, exposed to full medium (FM) or nutrient deprivation (ND) ± bafilomycin A1 (Baf) for 6 h. Cell lysates were analyzed for microtubule-associated protein 1 light chain 3 B (LC3) and β-actin. Quantified bands are expressed relative to FM. **(b) **Representative image of cells stably expressing mCherry-green fluorescent protein (GFP)-LC3B (tandem-LC3), exposed to FM/ND medium for 6 h ± Baf. (b)i Fluorescent labeling of autophagosomes using tandem-LC3 allows differentiation between autophagosomes, autolysosome formation and degradation. **(c-f) **Tandem-LC3 cells were exposed to FM/ND ± Baf for 1 to 16 h and fluorescence intensities were analyzed by flow cytometry. Histograms represent distribution of fluorescence intensities of GFP (c) or mCherry (e) after 6 h (upper rows) or 16 h (lower rows) (see Additional file 1 for histograms of FM + Baf). Diagrams show mean fluorescence intensities (relative to fluorescence intensity under FM = 1) of GFP (d) and mCherry (f) after exposure to FM or ND ± Baf for 1 to 16 h.

Autophagic flux, that is, coupled autophagosome formation and degradation, can be inferred by comparing levels of cytosolic LC3-I and autophagosome membrane-bound LC3-II, in the absence and presence of lysosomal inhibitor [21]. Detected LC3 levels are referred to as steady state and cumulative, respectively. Here, lysosomal turnover of LC3 is demonstrated by western blot detection of LC3-I (cytosolic) and LC3-II (autophagosomal membrane), in the presence and absence of bafilomycin A1 (Baf), which deacidifies the (auto)lysosomes thereby inhibiting degradation [22] (Figure 1a). Nutrient deprivation (ND) conditions alone resulted in decreased levels of LC3-I and LC3-II, and addition of Baf increased LC3-I/II levels to a greater extent under ND conditions compared to full medium (FM). Notably, quantification of western blot data allows calculation of flux, however, results are of low resolution, as indicated by high standard error of the mean (SEM) (Figure 1a).

As an alternative approach, we sought to differentiate between autolysosome formation and degradation steps utilizing the difference in pH sensitivity between GFP and mCherry in live cells [23]. When fused to the autophagic marker LC3, GFP fluorescence is quenched upon fusion between autophagosome and lysosome due to the low lysosomal pH, while mCherry remains fluorescent until degradation by lysosomal proteases [18,24]. Cells were stably transfected with tandem-LC3. In live cells, high-resolution imaging demonstrated that at 6 h, ND conditions increased the levels of mCherry-positive autolysosomes compared to FM control conditions, with few apparent autophagosomes (mCherry-positive and GFP-positive; that is, yellow) (Figure 1b). The addition of Baf here resulted in the accumulation of colocalized GFP-positive and mCherry-positive autophagosomes and autolysosomes (yellow) (Figure 1b, i). These results indicate that autolysosome formation is rapid under both basal (FM) and induced (ND) autophagy conditions, and highlights the lack of phenotype using GFP-LC3 alone. However, high-resolution imaging of tandem-LC3 revealed variability between cells, that is, not all cells show the same level of accumulated autolysosomes (Figure 1b, white arrows), as described previously [25]. Moreover, imaging is restricted to low sample numbers with manual selection of cells, thus potentially skewing or failing to capture the variability of responses within populations.

To overcome the disadvantages described above, we utilized flow cytometry to quantify responses at the population level, an approach used previously to quantify autolysosome formation [26]. Changes to GFP and mCherry fluorescence intensities (Figure 1b, i) were detected in live cell populations, under either FM or ND conditions for 6 and 16 h. Histograms show the distribution of GFP (Figure 1c) and mCherry (Figure 1e) fluorescence intensities, respectively. In response to ND, GFP fluorescence intensity decreased to 80% after 6 h and to less than 50% after 16 h of treatment. In contrast, mCherry fluorescence intensity decreased to approximately 85% after 6 h and 80% after 16 h of treatment. Baf was included to block lysosomal degradation during both ND-induced autophagy (Figure 1c,e) and basal autophagy (FM) (Additional file 1). At 6 h, Baf maintained GFP and mCherry fluorescence intensity at levels slightly less than under FM conditions. At 16 h, Baf treatment resulted in both GFP and mCherry fluorescence intensities greater than under FM conditions. Hence, our approach allowed for quantification of both autolysosome formation and degradation in a highly specific manner, indicated by the block of GFP/mCherry turnover by addition of Baf.

We further quantified time-resolved changes of GFP and mCherry fluorescence levels in response to FM or ND ± Baf for 1 to 16 h (Figure 1d,f). Results are reported as fold increase or fold decrease relative to the intensity levels of basal FM steady-state autophagy conditions (that is, assigning fluorescence intensity of FM = 1), thereby reporting relative impact of different time points and conditions. In response to ND, GFP fluorescence intensity decreased in a linear manner over time (Figure 1d), whereas the decrease of mCherry fluorescence intensity slowed down following the 6 h time point (Figure 1f), indicating continuous vesicle fusion, with lysosomal degradation as the limiting step in autophagy. The decreases in both GFP-LC3 and mCherry-LC3 fluorescence intensities were significant after 1 h of exposure to ND conditions, underlining the sensitivity of flow-cytometry-based detection in contrast to western blotting (Figure 1a). Notably, although degradation steps could be efficiently blocked by addition of Baf, our assay does not rely on addition of lysosomal inhibitors to infer autophagic flux. Instead, the loss of GFP fluorescence intensity reports autolysosome formation and mCherry fluorescence intensity corresponds to autolysosome degradation, allowing direct readout of autophagic flux.

To exclude the possibility of proteasomal degradation of tandem-LC3, we used the specific proteasome inhibitor epoxomicin (Epox; 1 μM) under both FM and ND conditions (Additional file 2; high-resolution images are shown in Additional files 3 and 4). Under FM conditions, inhibition of the proteasome slightly suppressed GFP fluorescence at 1 to 5 h timepoints, indicating an initial weak activation of autophagy. Prolonged treatment (> 8 h) resulted in a detectable increase in GFP fluorescence, indicating an inhibition of autolysosome formation. No changes were detected for mCherry fluorescence, indicating that Epox did not impact autolysosome degradation. Importantly, under ND conditions, Epox did not affect GFP or mCherry fluorescence from 1 to 6 h, indicating that inhibition of proteasomal activity had no effect on degradation of tandem-LC3 under ND for the first 6 h. From timepoints 8 to 16 h GFP and mCherry fluorescence was increased, indicating an inhibitory effect of Epox on autophagic degradation. Thus, quantification of tandem-LC3 turnover by flow cytometry is specific for autophagic degradation, indicated by sensitivity to Baf and insensitivity to Epox treatment.

### Flow cytometry detection of endolysosomal activity using GFP-Rab7

**Figure 2:**
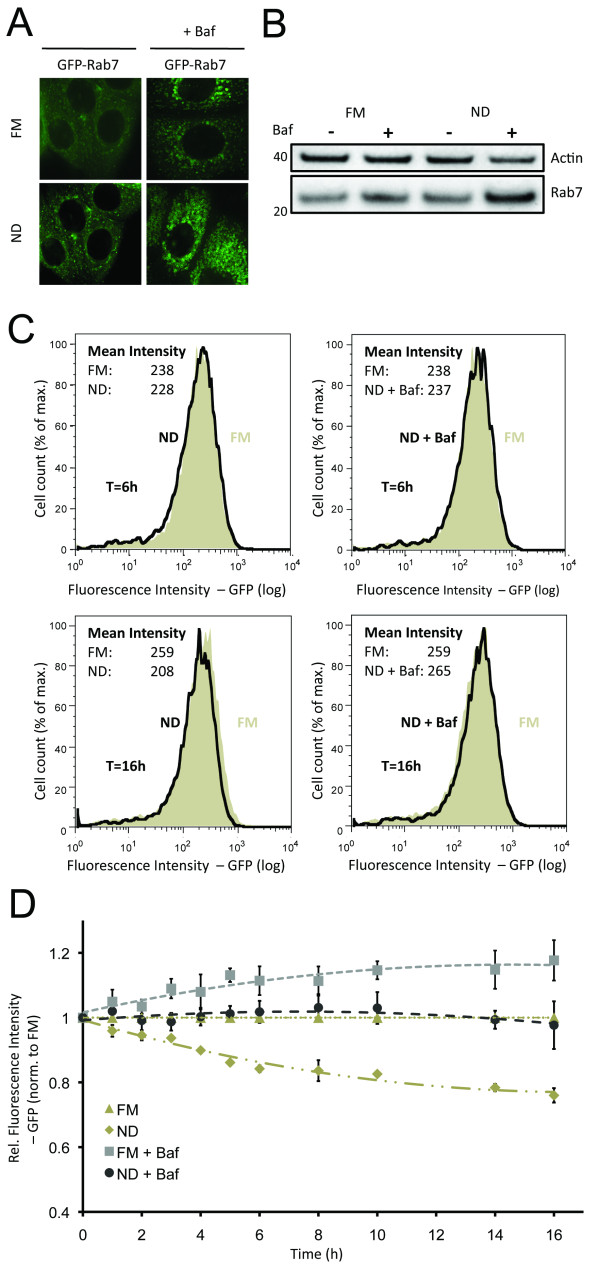
**Quantitative detection of endolysosomal activity using flow cytometry**. **(a) **Representative image of green fluorescent protein (GFP)-Rab7 cells, exposed to full medium (FM) or nutrient deprivation (ND) medium for 6 h ± bafilomycin A1 (Baf). **(b) **Western blot analysis of wild-type cells, exposed to FM or ND ± Baf for 6 h. Cell lysates were analyzed for Rab7 and β-actin. **(c) **Histograms show distribution of GFP-Rab7 fluorescence intensity after 6 h (upper row) or 16 h (lower row) incubation with FM or ND ± Baf. **(d) **Diagram represents mean fluorescence intensity (relative to fluorescence intensity under FM = 1) of GFP-Rab7 for 1 to 16 h incubation with FM/ND ± Baf.

The endolysosomal pathway is coupled to diverse cell mechanisms, including endocytosis, signal specificity and autophagy [12], as autophagosomes fuse with late endosomes during autolysosomal maturation [9]. To assess potential crosstalk between endolysosomal and autophagic degradation, we sought to utilize GFP-Rab7 [19,20] as an indicator of general changes in the endolysosomal degradative pathway. Through comparison of tandem-LC3 and GFP-Rab7 readouts it was possible to differentiate specific effects on the autophagic pathway from general changes in the endolysosomal degradation pathway. Analysis of GFP-Rab7 by high-resolution imaging indicated that, similar to LC3, endolysosomal flux can be inferred by comparing the number of Rab7 vesicles in presence and absence of Baf (Figure 2a). Under ND conditions low numbers of small GFP-Rab7 vesicles are detected. In response to Baf, the cytoplasm filled with larger GFP-Rab7 vesicles. Western blot analysis confirmed that endogenous Rab7 turnover is Baf sensitive under both FM and ND conditions (Figure 2b), with higher turnover under ND conditions. We further confirmed the specificity of Rab7 for the late endosomal and lysosomal compartments by cotransfecting stable GFP-Rab7 cells with either mCherry-Rab5 (early endosomal marker) or Lamp1-red fluorescent protein (RFP) (lysosomal marker). As expected, Rab7 localized to the Lamp1-RFP-positive lysosomal, rather than to the mCherry-Rab5 early endosomal compartments (Additional file 5). Analogous to GFP-LC3, we measured the decrease of GFP-Rab7 fluorescence intensity under FM and ND conditions (Figure 2c). ND increased the loss of GFP fluorescence intensity in a time-dependent manner, however with decreasing turnover rates after 6 h of incubation (Figure 2d). Similar to GFP-LC3, addition of Baf inhibited the decrease in GFP-Rab7 fluorescence intensity, but was not required to detect endolysosomal flux (Figure 2 and Additional file 1).

In summary, flow cytometry detection of tandem-LC3 and GFP-Rab7 allows for detection of autophagic and endolysosomal flux, respectively. Importantly, quantitative flux measurements are determined without the need for lysosomal inhibitors, as flux could be inferred by comparing FM to ND conditions. Changes in fluorescence intensity of GFP-LC3, mCherry-LC3 and GFP-Rab7 upon exposure to ND were already significant after 1 h of incubation (*P *< 0.05). However, to allow detection of minor drug-induced effects, 6 h incubations were utilized in subsequent experiments, as higher significant responses were detected (*P *< 0.001 for FM vs ND). Furthermore, normalization of responses allows for relative comparison of autophagic and endolysosomal responses, as well as condition-dependent and time-dependent impacts.

### Drug induction of autophagy differentially impacts autophagic and endolysosomal activities under FM and ND conditions

**Figure 3:**
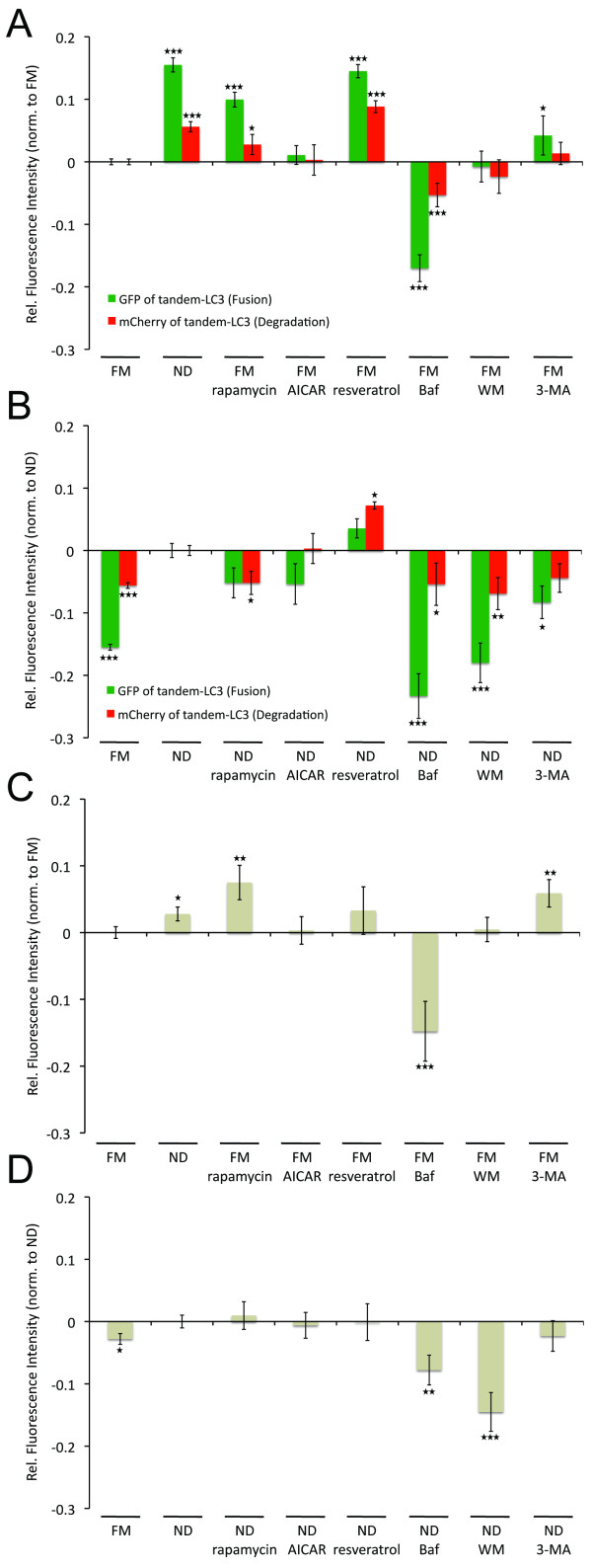
**Impact of autophagic regulators on autophagosomal fusion/degradation and endolysosomal turnover**. mCherry-green fluorescent protein (GFP) (tandem)-microtubule-associated protein 1 light chain 3 B (LC3) **(a,b) **or GFP-Rab7 **(c,d) **cells were exposed to either full medium (FM) (a,c) or nutrient deprivation (ND) (b,d) conditions ± respective drugs (rapamycin, 0.1 μM; AICAR, 200 μM; resveratrol, 100 μM; bafilomycin A1 (Baf), 0.1 μM; wortmannin (WM), 2.3 μM; 3-MA, 5 mM) for 6 h. Fluorescence intensities of tandem-LC3/GFP-Rab7 were detected by flow cytometry. Data was normalized as described in Materials and methods (including normalization to control (Ctr) constructs). Values represent fold changes in relation to the respective control condition (FM for (a,c) and ND for (b,d)). *P < 0.05, **P < 0.01, ***P < 0.001.

As a benchmark, we next examined the relative impact of reported autophagy modulating compounds, under conditions of basal and ND-induced autophagy (Figure 3). Values represent fold-change (for example, 0.10 = 10%) increase or decrease relative to either FM or ND control measurements, depending on indicated condition. An important consideration to the use of fluorescent proteins is the impact of cellular pH levels, protein synthesis, and compound autofluorescence. Thus, to compensate for non-specific changes to fluorescence intensities, experiments were performed under identical conditions in cells stably expressing control (Ctr)-tandem or Ctr-GFP. Depending on the stimulus, fluorescence intensities varied by up to 7% (results not shown). By correcting each data point for either tandem-LC3 or GFP-Rab7 to the respective control value, changes unrelated to autophagy were minimized (see Materials and methods for details). Although we report changes in fluorescence intensity of both GFP/mCherry, the corresponding ratio provided no additional insight, as differences cannot be captured (for example, simultaneous increase in GFP/mCherry, see Additional file 6).

Cells were treated with autophagy inducers rapamycin (inhibitor of mTOR; 0.1 μM) [27], AICAR (activator of AMPK; 200 μM) [28,29] or resveratrol (100 μM) [30] under either FM or ND conditions for 6 h. In addition, we tested the effect of three widely used autophagy inhibitors: Baf (0.1 μM), inhibiting lysosomal function, and wortmannin (WM; 2.3 μM) [31] and 3-methyladenine(3-MA; 5 mM) [32], two phosphoinositol 3 (PI3) kinase inhibitors which inhibit autophagy induction. See Additional files for high-resolution images of the respective drugs under FM (Additional file 3) and ND (Additional file 4) conditions, in presence and absence of Baf.

Rapamycin and resveratrol both induced a significant increase in autophagic activity compared to FM (Figure 3a). Notably, under FM conditions, resveratrol enhanced autolysosomal degradation to levels greater than induced by ND conditions. AICAR, which by activating AMPK inhibits mTOR [33], had no significant effect on autolysosome fusion or degradation. Similar to FM conditions, resveratrol further increased both autolysosome formation and degradation under ND conditions, with a more pronounced enhancing effect on autolysosomal degradation (Figure 3b). In contrast to FM conditions, rapamycin decreased both autolysosome formation and degradation under ND conditions. Rapamycin significantly increased endolysosomal flux under FM conditions, even above ND induced turnover (Figure 3c). However, rapamycin had no significant effect on endolysosomal activity under ND conditions (Figure 3d), indicating differential regulation for FM and ND conditions. Both AICAR and resveratrol had no significant effect on endolysosomal turnover (Figure 3c,d).

Notably, Baf was the only compound inhibiting autolysosomal formation and degradation events and endolysosomal turnover under both FM and ND conditions (Figure 3). Under FM conditions, WM and 3-MA had no inhibitory effect on autophagy, with 3-MA even slightly increasing formation of autolysosomes (Figure 3a). In contrast, both WM and 3-MA inhibited autolysosome formation and degradation under ND conditions, with WM being more efficient (Figure 3b). 3-MA also upregulated endolysosomal turnover under FM but had no effect under ND conditions (Figure 3c,d). In contrast, WM had no effects under FM, while inhibiting endolysosomal turnover under ND conditions, even more efficiently than Baf (Figure 3c,d). This indicates WM to be a more specific and potent inhibitor than 3-MA with respect to both autophagic and endolysosomal activity.

### High-content flow cytometry to identify small compounds enhancing basal autophagic flux

Defective autophagy is implicated in many diseases [1], and therefore the identification of drugs specifically modulating autophagic flux, without interfering with other endolysosomal processes, is of great translational interest. To that end, we modified the above workflow to screen the Prestwick Chemical Library, consisting of 1,120 FDA-approved compounds, for modulators of autophagy (Additional file 7).

**Figure 4:**
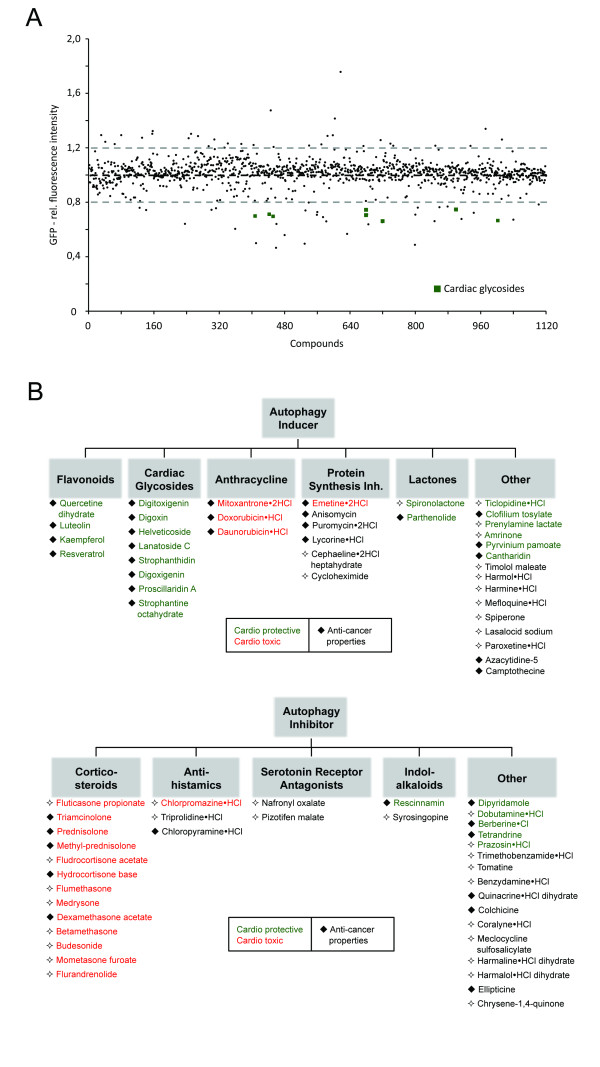
**Identification of potential activator and inhibitor of autophagy**. **(a) **Distribution of compounds. Fluorescence intensities are expressed relative to full medium (FM) conditions (fluorescence intensity under FM = 1). Each plate contained nutrient deprivation (ND) medium (relative fluorescence intensity = 0.55) and rapamycin (relative fluorescence intensity = 0.71) as positive and bafilomycin A1 (Baf) (relative fluorescence = 1.37) as negative controls. Compounds were considered as hits if fluorescence intensities were higher/lower than mean ± σ. **(b) **Primary hits identified by flow cytometry based autophagy screen. Autophagy inducers/inhibitors were grouped into respective classes. Color labeling (green/red) represents previously reported cardioprotective/cardiotoxic effects. Previously reported anti-cancer properties are indicated by filled squares. Respective PubMed database identification numbers (PMIDs) can be found in Additional file 9.

Using the 96-well plate format, drug impact was determined by measuring GFP-LC3 fluorescence intensity under basal (FM) autophagy conditions (Figure 4a). GFP-LC3 was used as a primary readout, since either upregulation or downregulation in autophagic activity would first be manifested in changed fluorescence intensity levels of GFP-LC3. Possible effects downstream of autolysosome formation are then identified in a secondary screen, using tandem-LC3 and GFP-Rab7 reporters, as well as LysoTracker Red (LTR) to assess lysosomal activity. Cells were incubated with drugs (10 μg/ml) in FM for 6 h. This early time point was determined sufficient to obtain significant differences without increasing the risk of long-term impacts, such as secondary effects influencing global autophagy or protein levels. Based on changes to GFP-LC3 fluorescence intensity we identified 38 potential inducers and 36 potential inhibitors of autolysosomal formation (Figure 4b), with a threshold applied at mean ± σ. Among these hits were compounds previously reported as regulators of autophagy, including autophagy inducers resveratrol [30] and camptothecin [34], as well as autophagy inhibitors colchicine [35] and quinacrine [36] (Figure 4b).

### Discovery and validation of cardiac glycosides as potent activators of autophagic activity

Remarkably, the 38 potential enhancers of autophagy included all of the 8 cardiac glycosides present in the Prestwick Chemical Library. Cardiac glycosides are commonly used in the clinical treatment of various heart conditions [37] and have recently emerged as potential cancer therapeutics [38]. Therefore, we selected the three most potent cardiac glycosides with clinical relevance (digoxin, strophanthidin, and digoxigenin) for validation and concentration-dependent analysis by measuring their effect on tandem-LC3, GFP-Rab7 and LTR (see Additional file 8 for concentration dependent analysis of additional primary hits).

**Figure 5:**
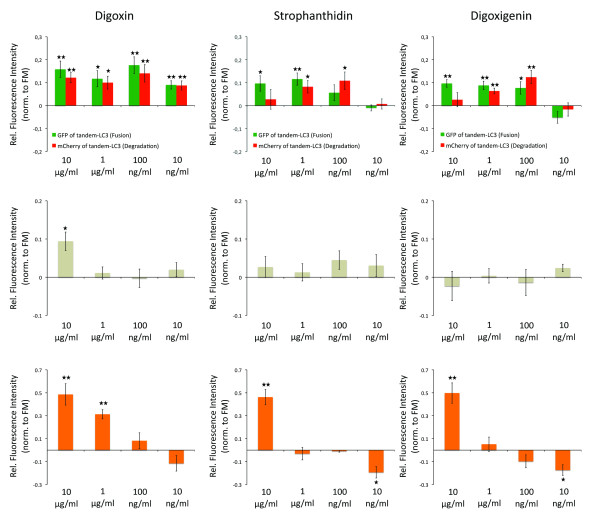
**Cardiac glycosides are novel and specific inducer of autophagic flux**. Diagrams showing autophagic activity (upper row), endolysosomal turnover (middle row) and lysosomal activity (bottom row), determined by flow cytometric quantification of fluorescence intensities of mCherry-green fluorescent protein (GFP) (tandem)-microtubule-associated protein 1 light chain 3 B (LC3), GFP-Rab7 and LysoTracker Red (LTR), respectively. Data was normalized as described in Materials and methods (including normalization to control (Ctr) constructs). Values represent fold changes in relation to full medium (FM) control condition. Drugs have been used at indicated concentrations under FM for 6 h. *P < 0.05, **P < 0.01, ***P < 0.001.

Drugs were retested at concentrations of 10 μg/ml, 1 μg/ml, 100 ng/ml and 10 ng/ml (Figure 5). Strophanthidin and digoxigenin induced autolysosome formation (loss in GFP fluorescence of tandem-LC3) at concentrations ranging from 10 μg/ml to 100 ng/ml; however, autolysosomal degradation (loss in mCherry fluorescence of tandem-LC3) was induced only at concentrations from 1 μg/ml to 100 ng/ml. Digoxin induced both autolysosome formation and degradation at concentrations ranging from 10 μg/ml to 10 ng/ml. At 10 μg/ml all drugs induced increased lysosomal activity (LTR), with digoxin additionally upregulating endolysosomal turnover (enhanced loss of GFP-Rab7 fluorescence). At 100 ng/ml no drug had a significant effect on endolysosomal turnover or lysosomal activity. Notably, although at higher concentrations strophanthidin and digoxigenin had a greater effect on autolysosome formation, autolysosome degradation rates were maximal with 100 ng/ml. Digoxin at 100 ng/ml maximally induced both formation and degradation of autolysosomes, indicating efficient enhancement of autophagic degradation. It is of clinical interest that at these concentrations, digoxin has been previously shown to induce apoptosis specifically in cancer cells and comparable concentrations can be found in plasma of cardiac patients [39].

**Figure 6:**
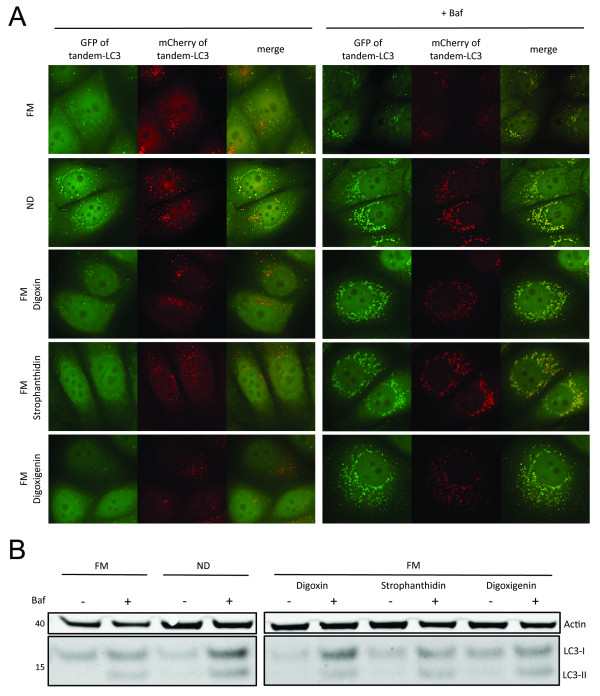
**Confirmation of autophagic flux induction by cardiac glycosides**. **(a) **Representative images of mCherry-green fluorescent protein (GFP)-microtubule-associated protein 1 light chain 3 B (LC3) cells; exposed to full medium (FM), nutrient deprivation (ND), FM + digoxin, strophanthidin or digoxigenin (each at 100 ng/ml) in presence/absence of bafilomycin A1 (Baf) for 6 h. **(c) **Wild-type cells were incubated with FM, ND, FM + digoxin, strophanthidin or digoxigenin (each at 100 ng/ml) in presence/absence of Baf for 6 h. Cell lysates were analyzed for LC3 and β-actin levels by western blotting.

We further confirmed the induction of autophagic flux by high-resolution microscopy and western blotting. At 100 ng/ml digoxin, strophanthidin, and digoxigenin all increased the Baf-dependent accumulation of GFP-LC3-positive autophagosomes compared to control conditions (Figure 6a). Likewise, all drugs increased the turnover of endogenous LC3-I and LC3-II as revealed under Baf treatment (Figure 6b). All approaches identified digoxin as the more potent inducer of autophagy than strophanthidin and digoxigenin.

## Discussion

Autophagy is of a highly dynamic nature and its interactions with the endolysosomal pathway are complex [9-12]. Therefore, analysis of autophagic activity requires detection of multiple pathway activities, including autophagosome formation and degradation, endolysosomal turnover and lysosomal degradative capacity. Here, we applied automated flow cytometry to quantitatively measure temporal, conditional, and drug-induced impacts on each of these individual steps. Key to our approach was the population sampling of single live cells, which generated multiparametric datasets amenable to statistical analysis. Specifically, single cell discrimination of pH quenching of GFP fluorescence of tandem-LC3 reported autolysosome formation, while loss of mCherry fluorescence of tandem-LC3 reported autolysosomal degradation. By comparing GFP and mCherry fluorescence intensities to the respective control conditions, autophagic flux was inferred without the need for lysosomal inhibitors (Figure 1). Furthermore, we established GFP-Rab7 turnover as a robust indicator for general changes in endolysosomal activity (Figure 2), allowing for the distinction between specific autophagic and general endolysosomal activity. Mean fluorescence intensities were sampled for both tandem-LC3 and GFP-Rab7, and outperformed western blot quantification in terms of sensitivity and accuracy (Figures 1 and 2). Thereby, our assay allows for comparison of multiple autophagy parameters, with respect to concentration, temporal, and conditional dependencies. Moreover, by using CMV promoter based reporter systems and normalization steps (Ctr-tandem/Ctr-GFP), we further assure the specificity of this approach to quantify autophagic activity independent of transcriptional or drug-induced off-target effects.

**Figure 7:**
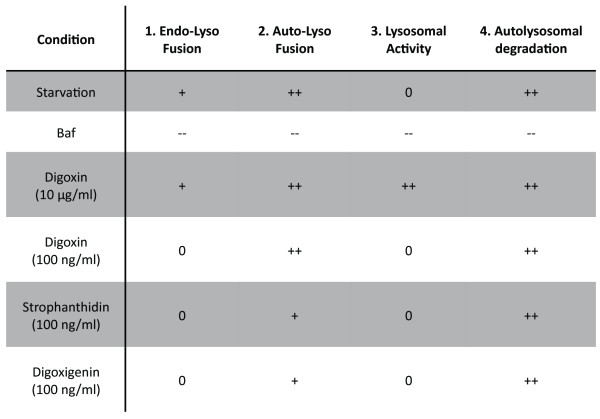
**Drug profiling by multiparametric quantitative analysis of autolysosomal degradation pathways**. Figure shows parameters for autolysosomal degradation pathways obtained by flow cytometry based screening of green fluorescent protein (GFP)-Rab7, GFP/mCherry (of mCherry-GFP (tandem)-microtubule-associated protein 1 light chain 3 B (LC3)) and LysoTracker Red (LTR).

Overall, combining multiparametric flow cytometry with high-content markers for autolysosomal degradation pathways improves standard screening methods [14-16] due to reduced risk of potential off-target effects by the addition of lysosomal inhibitors. Moreover, our approach outperforms current flow-cytometry-based autophagy assays [26,40] through quantification of both autolysosomal formation and degradation as well as capturing changes in the endolysosomal pathway. The ability of our assay to facilitate the identification of specific regulators of autophagy is highlighted by the drug-specific chart of activities of different autophagic steps, obtained by individually monitoring the involved autolysosomal degradation pathways (Figure 7). The importance of such an approach was apparent in our initial benchmark where we analyzed compounds that are widely used to inhibit and activate autophagy. We tested the effect of these compounds under conditions of both basal (FM) and activated (ND) autophagy. Activation of AMPK by AICAR had no effect on autophagy. This is in line with previous studies, reporting AMPK-independent effects of AICAR that block autophagy [41,42].

Strikingly, we found that drug impact can be strongly dependent on the underlying condition, with drugs having opposing effects if applied under FM or ND conditions. Rapamycin (Figure 3), commonly applied to induce autophagy [27], enhanced autophagy under FM, but had a surprising inhibitory effect under ND conditions. ND is well established to strongly inhibit mTOR [43]. Thus, the addition of rapamycin under ND conditions may not lead to additional mTOR inhibition, but instead inhibit autophagy by non-specific effects. Indeed, a previous report suggested mTOR independent inhibitory effects on autophagy for increased concentrations of rapamycin (> 250 nM) [16]. In contrast, 3-MA (Figure 3), commonly used as an autophagy inhibitor [32], decreased ND-induced autophagy, but activated autophagy if applied under FM conditions. Autophagy is regulated by two PI3Ks; while class III PI3K is required for the induction of autophagy, class I PI3K negatively regulates autophagy. Depending on the condition, inhibition of PI3K by WM or 3-MA might therefore have either inhibitory or activating effects [44]. Furthermore, our results suggest both negative and positive regulation of endolysosomal activity by PI3K, similar to autophagic regulation. Thus, the sensitivity of our approach allows for the comparison of condition-dependent and relative potencies of autophagy modulators. Here, we showed condition-dependent effects of rapamycin and identified Baf as the most potent inhibitor under both FM and ND, followed by WM being more specific and potent than 3-MA.

We subsequently identified novel modulators of autophagy by screening 1,120 FDA approved, bioactive small compounds (Figure 4), demonstrating the translational potential of our approach. GFP-LC3 was used as a primary readout for autolysosome formation in live cells, as previously described by Shvets *et al*. [26]. Selected hits were then subjected to detailed analysis by monitoring effects on tandem-LC3, GFP-Rab7, and LTR. Thereby, we were able to identify defects downstream of autolysosome formation and confirm specific induction of the autophagic degradation pathway.

Applying this workflow, about 3% of screened compounds were classified as potential activators and 3% as potential inhibitors of autophagy (Figure 4b). Some of these hits have previously been reported as autophagic regulators, including resveratrol [30], camptothecin [34], colchicine [35] and quinacrine [36], demonstrating the accuracy of our approach for compound screening. Strikingly, the novel hits of our screen contained a family of eight compounds, cardiac glycosides, which are commonly used to treat heart failure [37] and more recently as cancer therapeutics [38]. These findings demonstrate the potential of high-content autophagy screening for identifying dual-purpose compounds with the goal of minimizing damage to essential, non-proliferating cells, while targeting proliferating cancer cells. For three cardiac glycosides, digoxin, strophanthidin and digoxigenin, we determined the optimal concentrations to specifically induce autolysosome formation and degradation, without affecting general endolysosomal activity (Figure 5). We further confirmed activation of autophagy by western blotting and imaging approaches (Figure 6). Cardiac glycosides are known to inhibit Na^+^-K^+^-ATPases [38], leading to increased calcium levels and thereby giving a possible mechanism for their effect on autophagy. Calcium can upregulate autophagy [21,45] and modulators of calcium are prominent among compounds identified in other autophagy screens [14,15,46]. Cardiac glycosides have been suggested for cancer therapy, due to their potential to induce tumor specific cell death [38]. It is remarkable that many of the identified autophagy activators have both anti-cancer and cardioprotective properties (Figure 4b and Additional file 9), indicating potential drugability of autophagic cell death-associated mechanisms. Conversely, many of the reported autophagy inhibitors induce cardiotoxicity and have anti-cancer properties. The role of autophagy inhibition in the efficiency of cell death induction during chemotherapy warrants further study [47]. Our study demonstrates the suitability of high-content screening for the characterization of localized-drug impact on autophagy. Future work might employ inducible expression systems, potentially also including additional sensors, such as GFP-p62 [48], thereby further increasing the sensitivity of our approach and facilitating the portability to other cell types.

## Conclusions

In summary, we present a multiparametric screening approach, validated against common imaging and biochemical assays, which allows for quantitative measurements of the entire autolysosomal pathway, independent of lysosomal inhibitors. The ability to measure relative impacts on different pathway events revealed striking conditional differences between the most commonly used drug modulators of autophagy. In addition, this approach was highly scalable, allowing for quantitative drug screening of 1,120 small compounds. From within a total number of 74 hits, cardiac glycosides were identified and 3 were further validated as novel inducers of autophagy. Thus high content autophagy screening is effective for identifying drugs of interest for highly relevant disease type and thereby suggesting clear treatment strategies for *in vivo *confirmation.

## Methods

### Cell culture

MCF-7 breast cancer cells (Cell Lines Services, Heidelberg, Germany) were cultivated in FM, consisting of Dulbecco's modified Eagle medium (DMEM) (Invitrogen, Karlsruhe, Germany) supplemented with 10% fetal calf serum, non-essential amino acids, Glutamax (Invitrogen) and penicillin/streptomycin/amphotericin, at 37°C with 5% CO_2_. Stable cell lines (GFP-LC3, tandem-LC3, GFP-Rab, GFP and mCherry-GFP), were generated via transfection (Effectene; Qiagen, Hilden, Germany) with the respective construct and selection through addition of G418 (Invitrogen) at 500 μg/ml. Single cell colonies were selected and stable cell lines were cultured in the presence of 100 μg/ml G418. Transient transfection (mCherry-Rab5 and Lamp1-RFP) was carried out with Effectene (Qiagen). Cells were incubated in full medium (FM) or nutrient deprivation (ND) conditions as indicated. Cells were exposed to ND conditions by replacing FM with modified Krebs-Henseleit balanced salt solution (110 mM NaCl, 4.7 mM KCl, 1.2 mM KH_2_PO_4_, 1.25 mM MgSO_4_, 1.2 mM CaCl_2_, 25 mM NaHCO_3_, 15 mM glucose, 20 mM 4-(2-hydroxyethyl)-1-piperazineethanesulfonic acid (HEPES), pH 7.4).

### Construction of expression vectors

To generate mCherry-GFP-LC3B, LCB3 CDS was amplified from pEGFP-LC3 [24] inserted into pmCherry-C1. Subsequently enhanced GFP (EGFP) was inserted, in frame, between mCherry and LC3B. Rab5a (DKFZ clone repository, NM_004162) was amplified and inserted into pmCherry-C1 to generate mCherry-Rab5.

### Flow cytometry

Cells were cultured in 96-well plates at a density of 25,000 cells/well. For flow cytometric analysis, culture medium was removed and cells were incubated in 50 μl Trypsin-ethylenediaminetetraacetic acid (EDTA) (Invitrogen) for 5 min. Trypsin was inactivated by addition of 150 μl of ice-cold Krebs-Henseleit with 1% bovine serum albumin (Sigma-Aldrich, Munich, Germany). Flow cytometric analysis was carried out with a modified Beckman-Coulter (Krefeld, Germany) FC500MPL, allowing direct sampling from 96-well plates and simultaneous excitation and detection of green (488 nm) and red (561 nm) fluorescent proteins. At least 1,000 events were collected for each well. Color compensation was carried out for multicolor detection using matched single fluorescent proteins compensation controls. To minimize non-specific compound effects, each experiment was carried out in parallel with both the sensor construct and the respective fluorescent protein alone, that is, mCherry-GFP (Ctr-tandem) or GFP (Ctr-GFP). For each sample, cell number and mean fluorescence intensity were reported. All measurements were normalized to FM conditions and then corrected for unspecific changes by normalization to respective Ctr constructs, measured under identical conditions. The mean of the normalized values was expressed as fold changes to either FM or ND, as indicated. Positive values indicate activation and negative values indicate inhibition. Each experiment was carried out at least three times independently.

For staining of lysosomes, cells were incubated with LTR (Invitrogen) at a concentration of 50 nM for the last 30 min of treatment, washed once with the respective medium and then processed for analysis.

### Small compound screen

Stable GFP-LC3 cells were incubated with small compounds of the Prestwick Chemical library (Prestwick Chemical, Illkirch, France) at a concentration of 10 μg/ml in FM for 6 h. Rapamycin (0.1 μM) and ND were used as positive controls, and Baf (0.1 μM) as negative control. Controls were randomly distributed over first/last column and controlled for edge effects. Analysis by flow cytometry was carried out as described above. Each experiment was carried out in duplicate and compounds were excluded if duplicates differed more than 10%.

### Reagents

Bafilomycin A1 (Baf), rapamycin, wortmannin (WM), 3-MA, AICAR, resveratrol, and epoxomicin were purchased from Calbiochem (Darmstadt, Germany). Digoxin, digoxigenin, strophanthidin, doxorubicin, daunorubicin and mitoxantrone were purchased from Sigma-Aldrich. Drugs were diluted in dimethylsulfoxide (DMSO).

### Statistical analysis

Statistical significance was determined using a one-tailed Student's t test. Values are expressed as mean ± SEM.

### Microscopy

Cells were grown on iBidi 8-well slides (40,000 cells/well) and treated as indicated. High-content imaging was carried out using a DeltaVision RT deconvolution microscope (Applied Precision, Issaquah, WA, USA) equipped with a 60 × oil immersion objectives and a CCD digital camera (Hamamatsu, Herrsching, Germany). Images were deconvolved to maximize spatial resolution and processed using ImageJ software [49]. Images shown are maximum projections of Z stacks of representative cells (selected from at least three independent experiments).

### Immunoblotting

Cells were cultured in 6-well plates (400,000 cells/well) and treated as indicated. After indicated time points, whole cell lysates were prepared with radioimmunoprecipitation assay (RIPA) lysis buffer (Upstate, Charlottesville, VA, USA) containing Protease Inhibitor Cocktail (Roche, Mannheim, Germany). Protein concentrations were measured by Coomassie assay (Sigma-Aldrich), adjusted to obtain equal loading and mixed with NuPage sample buffer master mix (NuPage LDS buffer and reducing reagent, Invitrogen). Samples were then separated on 12% NuPage Bis-Tris gels (Invitrogen) and transferred to nitrocellulose membranes using the iBlot dry blotting system (Invitrogen). Immunodetection was carried out using primary antibodies against β-actin (Abcam, Cambridge, UK), LC3B (Cell Signaling, Danvers, MA, USA), and Rab7 (Cell Signaling). Membranes were prepared with horseradish peroxidase (HRP)-linked secondary antibodies (Cell Signaling) and chemiluminescence was detected using a Chemiluminescence Detection System (Intas, Göttingen, Germany). Immunoblots shown are representative of at least three independent experiments. Quantification of immunoblots (Figure 1a) was performed on three independent samples using ImageJ software [49]. Intensity of respective bands (LC3-II) were quantified and normalized to the loading control (β-actin).

## Authors' contributions

All authors participated in the design of the study. PH performed all experiments and performed the statistical analysis. All authors wrote, read and approved the final manuscript.

## Supplementary Material

Additional file 1 **Histograms of mCherry-green fluorescent protein (GFP) (tandem)-microtubule-associated protein 1 light chain 3 B (LC3) and GFP-Rab7 measurements**. Tandem-LC3 **(a,b) **or GFP-Rab7 **(c) **cells were exposed to full medium (FM), nutrient deprivation (ND) or FM + bafilomycin A1 (Baf) conditions and fluorescence intensities were analyzed by flow cytometry. Histograms represent distribution of fluorescence intensities of GFP (a) or mCherry (b) of tandem-LC3 and GFP-Rab7 (c) after 6 h (upper rows) or 16 h (lower rows) incubation with FM, ND or FM + Baf. Click here for file

Additional file 2 **Effect of proteasomal inhibition on degradation of mCherry-green fluorescent protein (GFP) (tandem)-microtubule-associated protein 1 light chain 3 B (LC3)**. Tandem-LC3 cells were exposed to full medium (FM)/nutrient deprivation (ND) ± epoxomicin (Epox; 1 μM) for 1 to 16 h and fluorescence intensities were analyzed by flow cytometry. Diagrams show mean fluorescence intensities (relative to fluorescence intensity under FM = 1) of GFP **(a) **and mCherry **(b) **after exposure to FM or ND ± Epox for 1 to 16 h. Click here for file

Additional file 3 **Effect of autophagic regulators on localization of mCherry-green fluorescent protein (GFP) (tandem)-microtubule-associated protein 1 light chain 3 B (LC3) under full medium (FM) conditions**. Representative images of cells stably expressing tandem-LC3. Cells were exposed to FM and respective autophagy regulators in presence/absence of bafilomycin A1 (Baf) for 6 h. Click here for file

Additional file 4 **Effect of autophagic regulators on localization of mCherry-green fluorescent protein (GFP) (tandem)-microtubule-associated protein 1 light chain 3 B (LC3) under nutrient deprivation (ND) conditions**. Representative images of cells stably expressing tandem-LC3. Cells were exposed to ND and respective autophagy regulators in presence/absence of bafilomycin A1 (Baf) for 6 h. Click here for file

Additional file 5 **Rab7 is a specific marker for late endosomal/lysosomal compartments**. Representative images of stable Rab7-green fluorescent protein (GFP) cells, cotransfected with Lamp1-red fluorescent protein (RFP) **(a) **or mCherry-Rab5 **(b)**. Cells were exposed to full medium (FM) or nutrient deprivation (ND) ± bafilomycin A1 (Baf) for 6 h. Click here for file

Additional file 6 **Ratio of green fluorescent protein (GFP)/mCherry of mCherry-GFP (tandem)-microtubule-associated protein 1 light chain 3 B (LC3)**. Ratio of relative fluorescence intensities of GFP/mCherry (of tandem-LC3), as determined in Figure 3. Click here for file

Additional file 7 **Screening approach for novel regulators of autophagic activity**. Green fluorescent protein (GFP)-microtubule-associated protein 1 light chain 3 B (LC3) cells were plated in 96-well format and incubated in full medium (FM) with small compounds (10 μg/ml) for 6 h. Experiments were carried out in duplicates and cells were analyzed by flow cytometry for fluorescence intensities of GFP-LC3. Selected hits were then run through secondary analysis by multiparametric analysis of lysosomal degradation pathways, quantification of mCherry-GFP (tandem)-LC3, GFP-Rab7 (including normalization to control (Ctr) constructs) and LysoTracker Red (LTR) at various concentrations. Hits were validated by biochemical/imaging analysis. Click here for file

Additional file 8 **Concentration-dependent effects of anthracyclines on autolysosomal degradation pathways**. Diagrams showing autophagic activity (upper row), endolysosomal turnover (middle row) and lysosomal activity (bottom row), determined by flow cytometric quantification of fluorescence intensities of mCherry-green fluorescent protein (GFP) (tandem)-microtubule-associated protein 1 light chain 3 B (LC3), GFP-Rab7 and LysoTracker Red (LTR), respectively. Data was normalized as described in Materials and methods (including normalization to control (Ctr) constructs). Values represent fold-changes in relation to full medium (FM) control condition. Drugs have been used at indicated concentrations under FM for 6 h. *P < 0.05, **P < 0.01, ***P < 0.001. Click here for file

Additional file 9 **List of compounds classified as hits by primary screen**. PubMed database identification numbers (PMIDs) of previously reported cardioprotective/cardiotoxic effects and anti-cancer properties as stated in Figure 4. Click here for file
