# Supplementary figures and images for: Concurrent detection of autolysosome formation and lysosomal degradation by flow cytometry in a high-content screen for inducers of autophagy
Source: BMC Biol. 2011 Jun 2;9:38. doi: 10.1186/1741-7007-9-38 (PMC3121655; doi:10.1186/1741-7007-9-38)

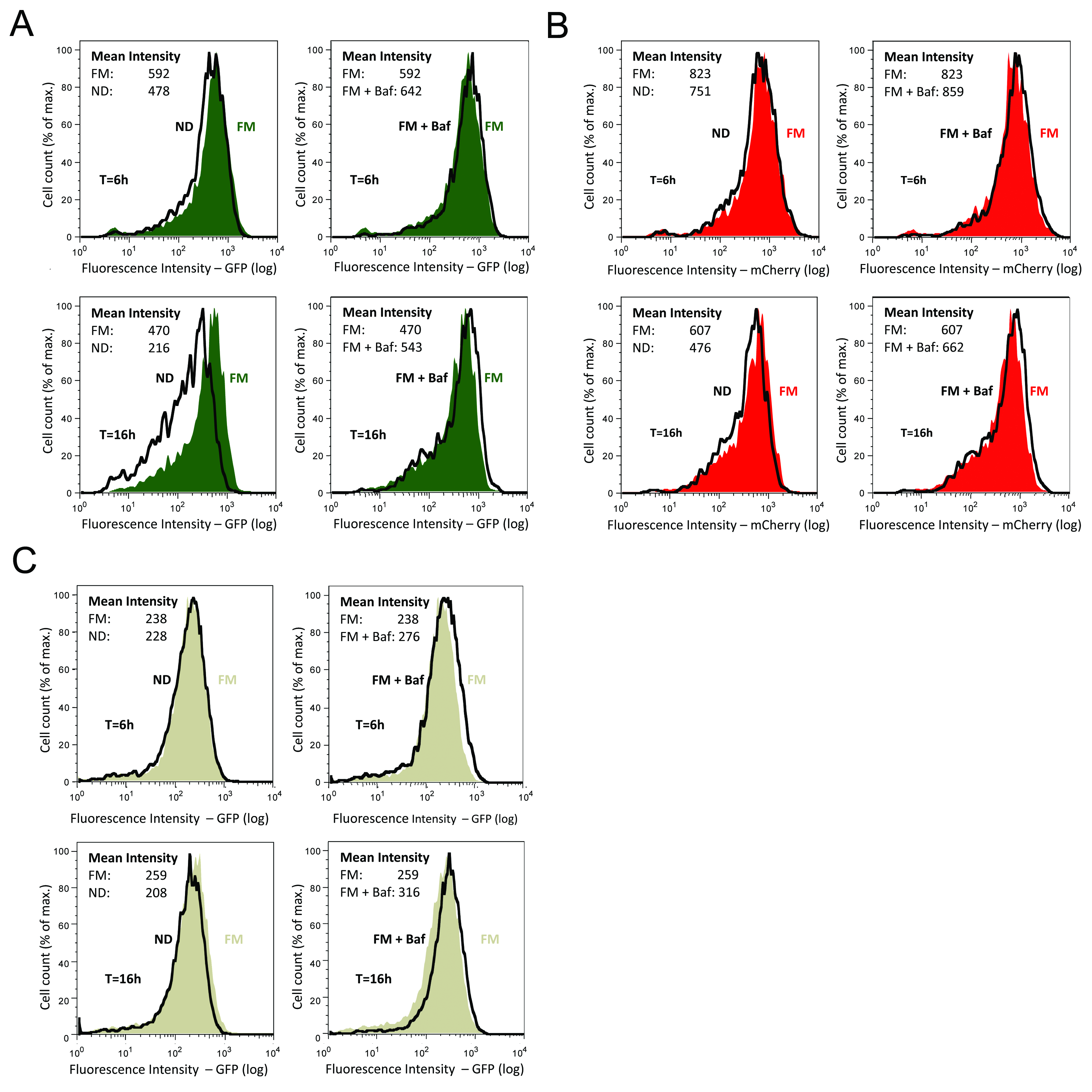

Supplement: Additional file 1 — Histograms of mCherry-green fluorescent protein (GFP) (tandem)-microtubule-associated protein 1 light chain 3 B (LC3) and GFP-Rab7 measurements. Tandem-LC3 (a,b) or GFP-Rab7 (c) cells were exposed to full medium (FM), nutrient deprivation (ND) or FM + bafilomycin A1 (Baf) conditions and fluorescence intensities were analyzed by flow cytometry. Histograms represent distribution of fluorescence intensities of GFP (a) or mCherry (b) of tandem-LC3 and GFP-Rab7 (c) after 6 h (upper rows) or 16 h (lower rows) incubation with FM, ND or FM + Baf. [file 1741-7007-9-38-S1.TIFF]

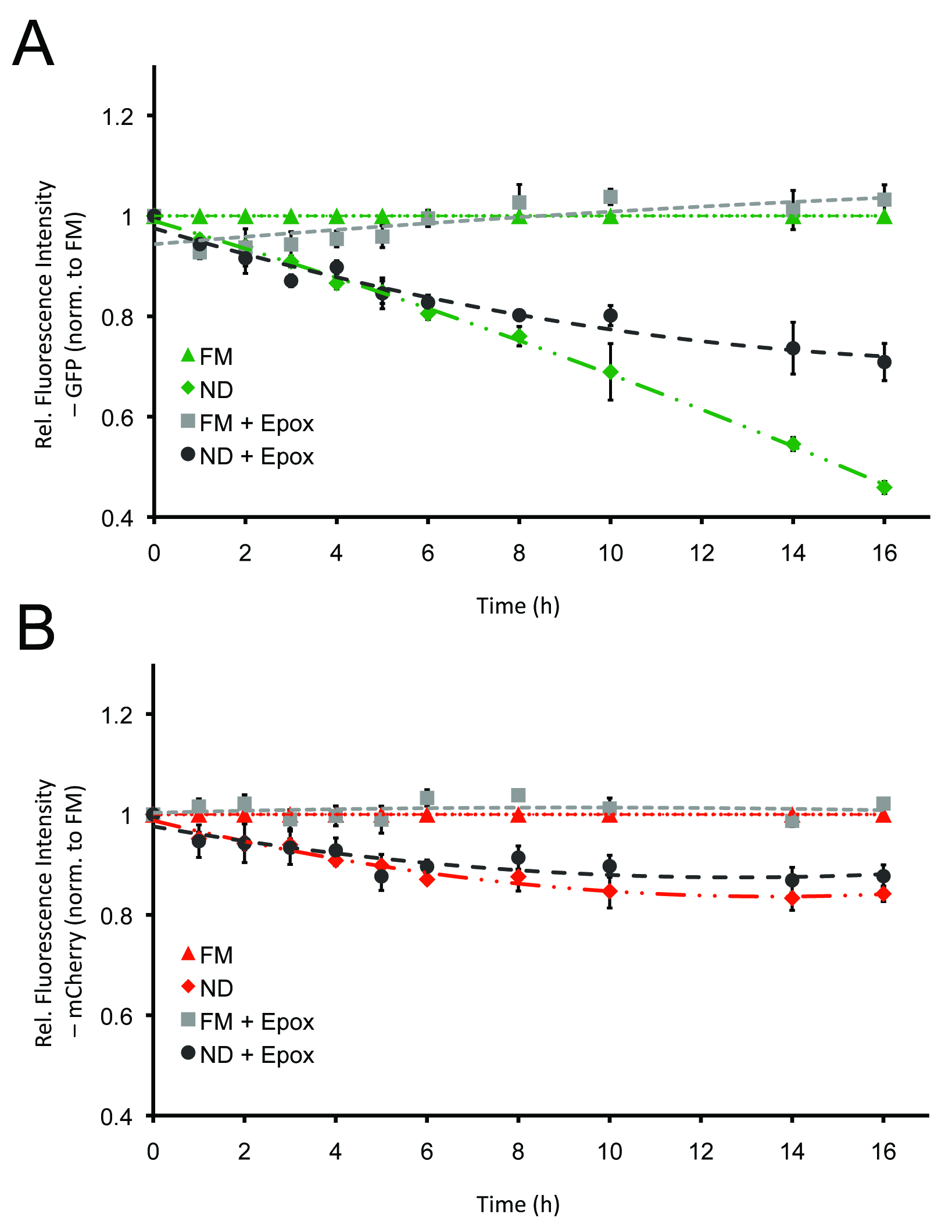

Supplement: Additional file 2 — Effect of proteasomal inhibition on degradation of mCherry-green fluorescent protein (GFP) (tandem)-microtubule-associated protein 1 light chain 3 B (LC3). Tandem-LC3 cells were exposed to full medium (FM)/nutrient deprivation (ND) ± epoxomicin (Epox; 1 μM) for 1 to 16 h and fluorescence intensities were analyzed by flow cytometry. Diagrams show mean fluorescence intensities (relative to fluorescence intensity under FM = 1) of GFP (a) and mCherry (b) after exposure to FM or ND ± Epox for 1 to 16 h. [file 1741-7007-9-38-S2.TIFF]

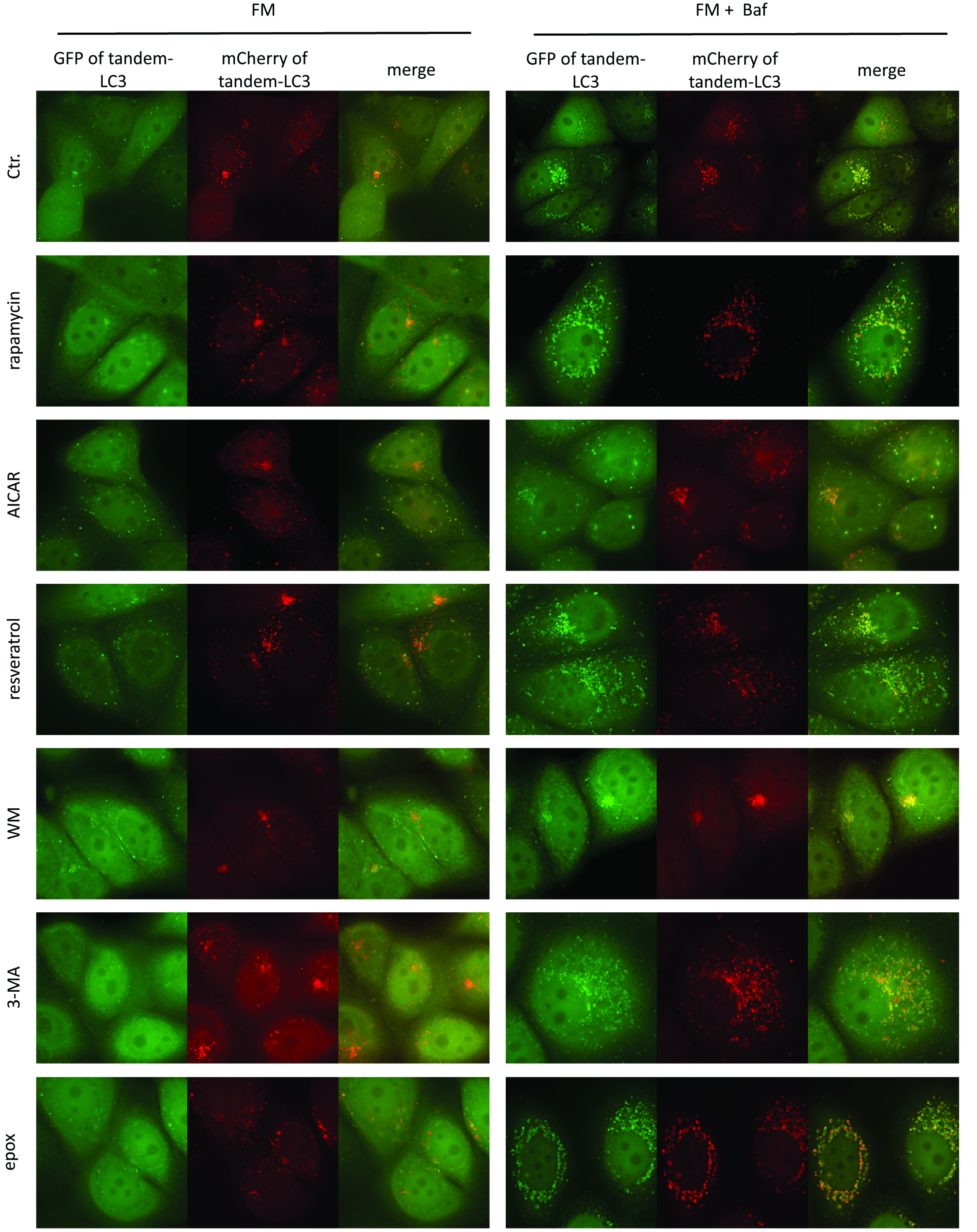

Supplement: Additional file 3 — Effect of autophagic regulators on localization of mCherry-green fluorescent protein (GFP) (tandem)-microtubule-associated protein 1 light chain 3 B (LC3) under full medium (FM) conditions. Representative images of cells stably expressing tandem-LC3. Cells were exposed to FM and respective autophagy regulators in presence/absence of bafilomycin A1 (Baf) for 6 h. [file 1741-7007-9-38-S3.TIFF]

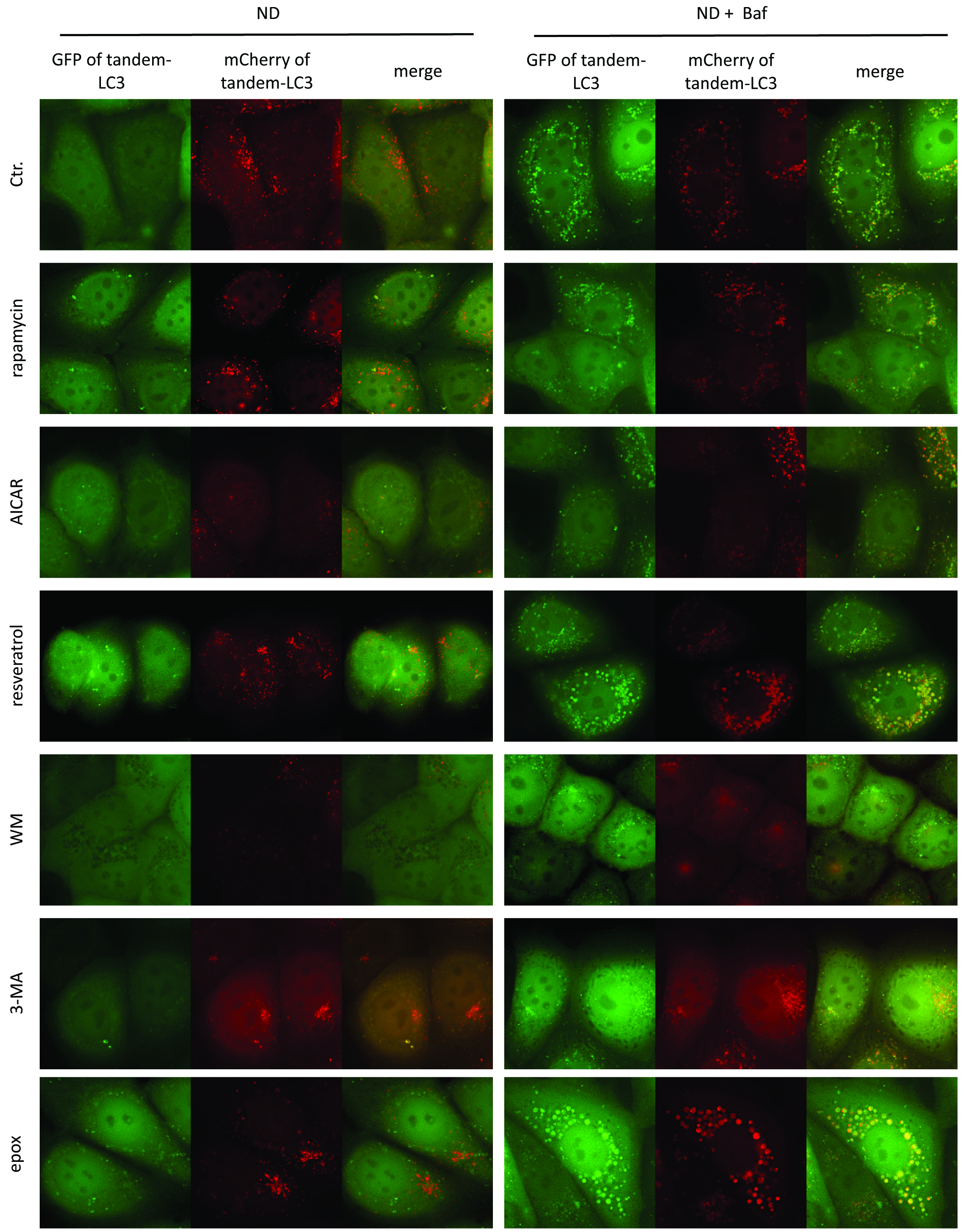

Supplement: Additional file 4 — Effect of autophagic regulators on localization of mCherry-green fluorescent protein (GFP) (tandem)-microtubule-associated protein 1 light chain 3 B (LC3) under nutrient deprivation (ND) conditions. Representative images of cells stably expressing tandem-LC3. Cells were exposed to ND and respective autophagy regulators in presence/absence of bafilomycin A1 (Baf) for 6 h. [file 1741-7007-9-38-S4.TIFF]

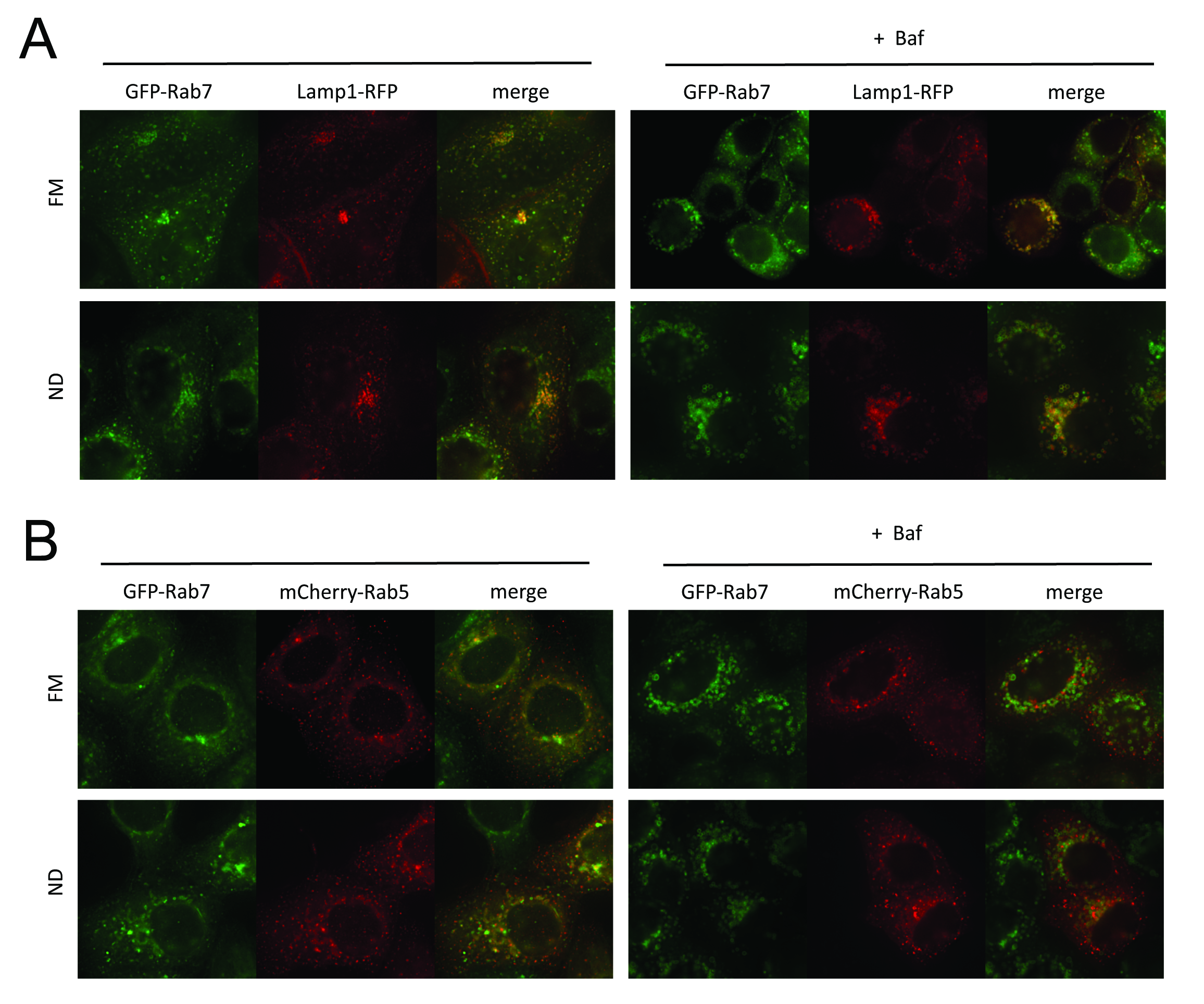

Supplement: Additional file 5 — Rab7 is a specific marker for late endosomal/lysosomal compartments. Representative images of stable Rab7-green fluorescent protein (GFP) cells, cotransfected with Lamp1-red fluorescent protein (RFP) (a) or mCherry-Rab5 (b). Cells were exposed to full medium (FM) or nutrient deprivation (ND) ± bafilomycin A1 (Baf) for 6 h. [file 1741-7007-9-38-S5.TIFF]

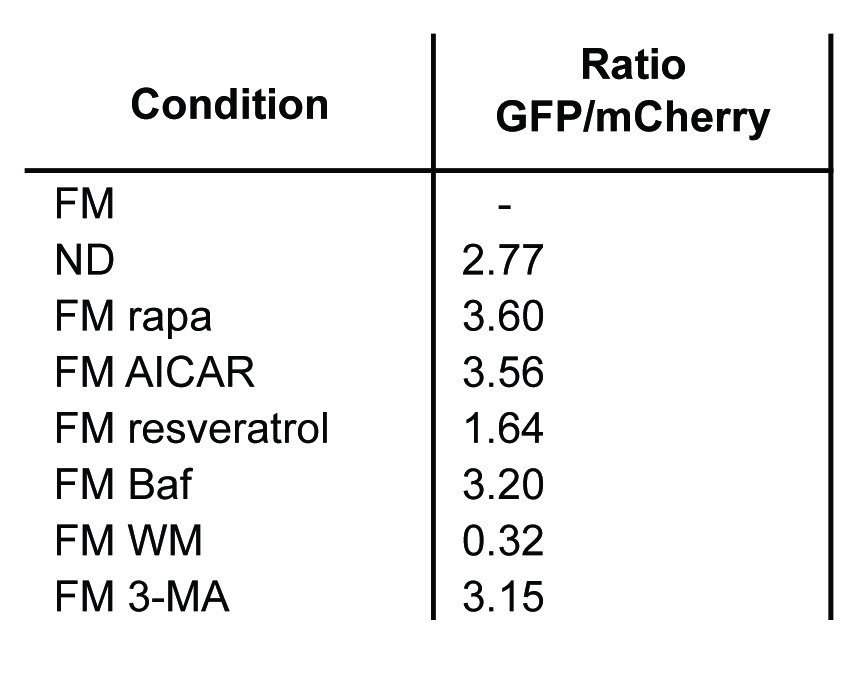

Supplement: Additional file 6 — Ratio of green fluorescent protein (GFP)/mCherry of mCherry-GFP (tandem)-microtubule-associated protein 1 light chain 3 B (LC3). Ratio of relative fluorescence intensities of GFP/mCherry (of tandem-LC3), as determined in Figure 3. [file 1741-7007-9-38-S6.TIFF]

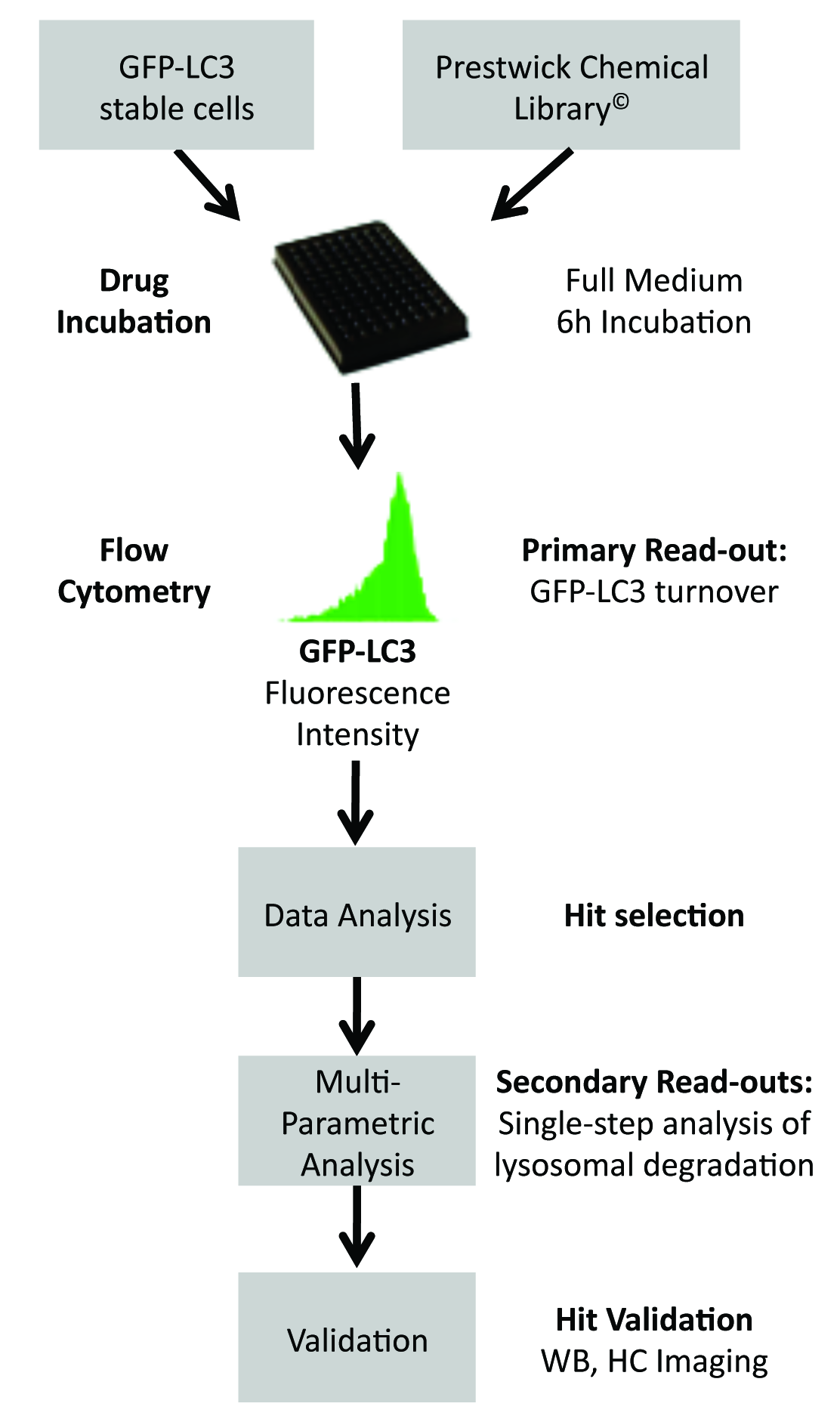

Supplement: Additional file 7 — Screening approach for novel regulators of autophagic activity. Green fluorescent protein (GFP)-microtubule-associated protein 1 light chain 3 B (LC3) cells were plated in 96-well format and incubated in full medium (FM) with small compounds (10 μg/ml) for 6 h. Experiments were carried out in duplicates and cells were analyzed by flow cytometry for fluorescence intensities of GFP-LC3. Selected hits were then run through secondary analysis by multiparametric analysis of lysosomal degradation pathways, quantification of mCherry-GFP (tandem)-LC3, GFP-Rab7 (including normalization to control (Ctr) constructs) and LysoTracker Red (LTR) at various concentrations. Hits were validated by biochemical/imaging analysis. [file 1741-7007-9-38-S7.TIFF]

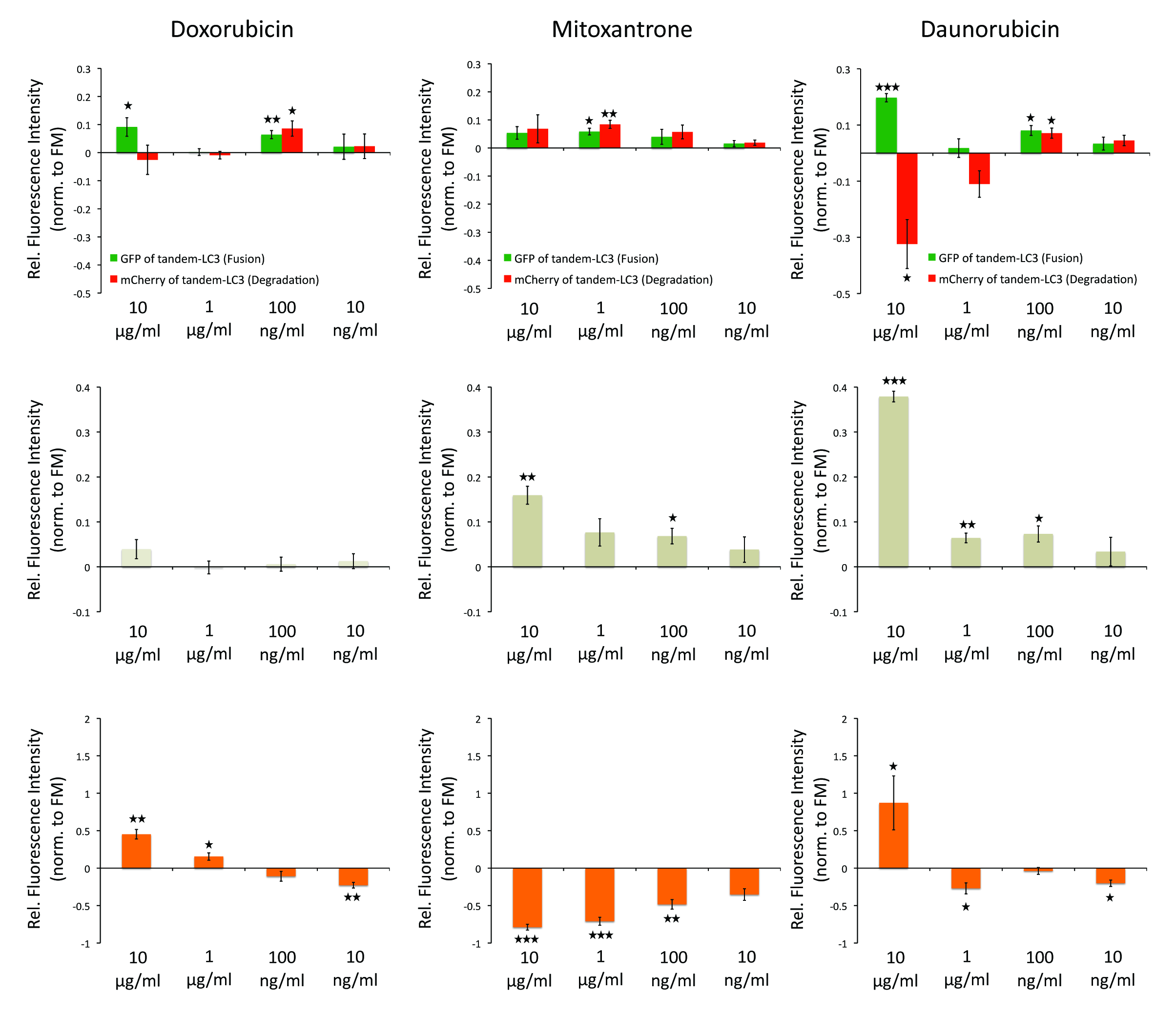

Supplement: Additional file 8 — Concentration-dependent effects of anthracyclines on autolysosomal degradation pathways. Diagrams showing autophagic activity (upper row), endolysosomal turnover (middle row) and lysosomal activity (bottom row), determined by flow cytometric quantification of fluorescence intensities of mCherry-green fluorescent protein (GFP) (tandem)-microtubule-associated protein 1 light chain 3 B (LC3), GFP-Rab7 and LysoTracker Red (LTR), respectively. Data was normalized as described in Materials and methods (including normalization to control (Ctr) constructs). Values represent fold-changes in relation to full medium (FM) control condition. Drugs have been used at indicated concentrations under FM for 6 h. *P < 0.05, **P < 0.01, ***P < 0.001. [file 1741-7007-9-38-S8.TIFF]

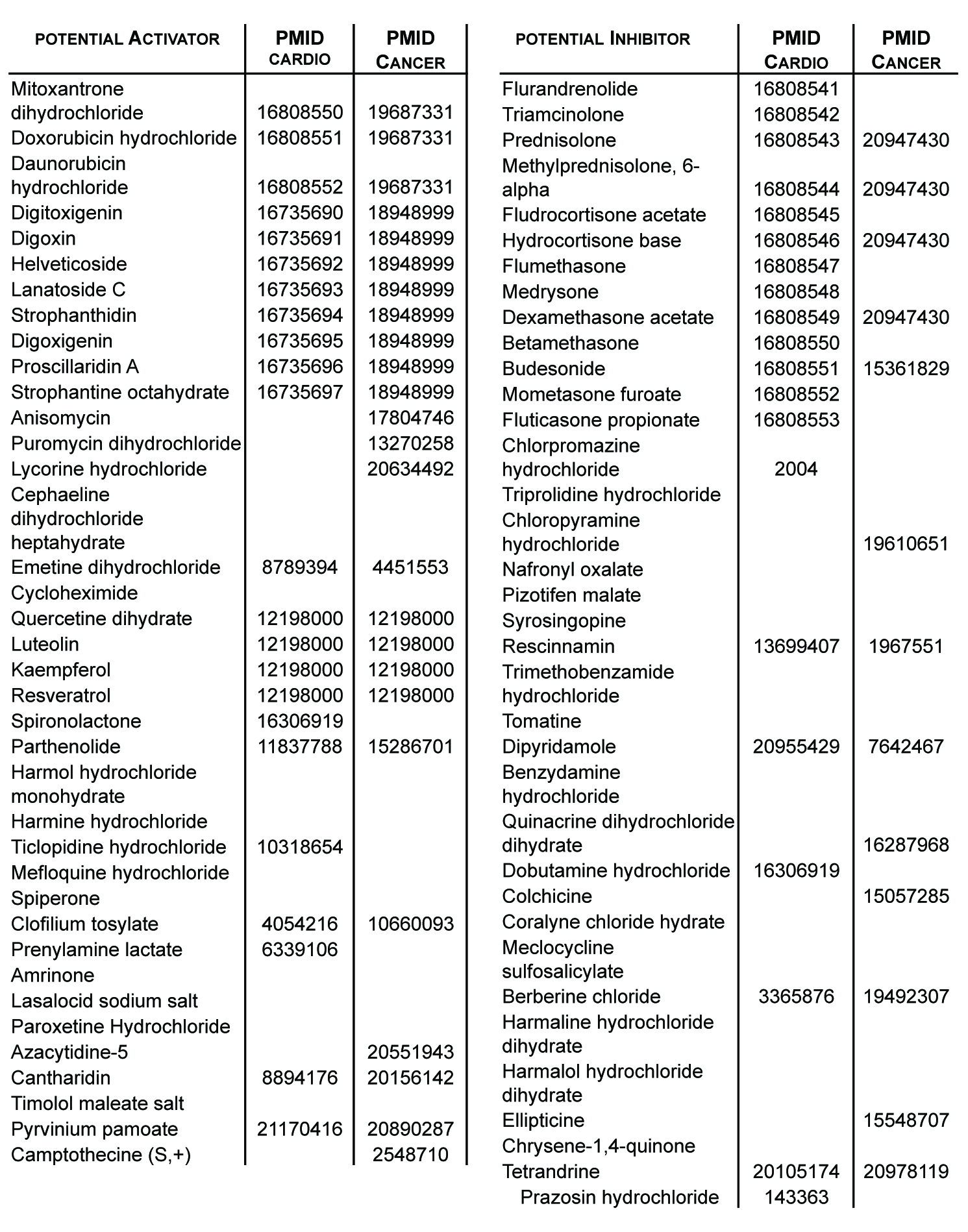

Supplement: Additional file 9 — List of compounds classified as hits by primary screen. PubMed database identification numbers (PMIDs) of previously reported cardioprotective/cardiotoxic effects and anti-cancer properties as stated in Figure 4. [file 1741-7007-9-38-S9.TIFF]
